# An Account of the Practice of One of the Physicians of the Westminster General Dispensary, and of the Western Dispensary, from the 20th of November, to the 20th of December, 1806

**Published:** 1807-01

**Authors:** S. Fothergill

**Affiliations:** Southampton Street, Strand


### *An Account of the Practice of one of the Physicians of the Westminster General Dispensary, and of the Western Dispensary, from the* 20*th of November, to the* 20*th of December,* 1806.

| | |
|---|---|
| ACUTE DISEASES. | |
| Rheumatism | 4 |
| Pleurisy | 2 |
| Scarlatina Anginosa | 3 |
| Synochus | 2 |
| Catarrh | 3 |
| Measles | 4 |
| Hooping-Cough | 2 |
| Small-pox | 1 |
| Enteritis | 1 |
| Acute Diseases of Infants | 8 |
| CHRONIC DISEASES. | |
| Pulmonary Consumption | 4 |
| Cough and Dyspnœa | 27 |
| Pleurodyne | 2 |
| Asthma | 1 |

Hæmoptoe

| | | | |
|---|---|---|---|
| Hæmoptoe - - | 3 | Palsy - - - | 2 |
| Epistaxis - - - | 1 | Inflammation of the Leg | 1 |
| Lumbago & Sciatica | 3 | Hernia - - - | 1 |
| Rheumatism - - | 6 | Aneurism - - | 1 |
| Cephalalgia and Vertigo | 4 | Hypochondriasis - | 2 |
| Asthenia - - | 8 | Hysteria - - - | 2 |
| Marasmus - - | 1 | Schirrus of the Breast | 1 |
| Tabes Mesenterica - | 3 | Uterine Hæmorrhage | 2 |
| Abdomen Tumidum - | 4 | Menorrhagia - - | 3 |
| Gastrodynia - - | 4 | Amenorrhœa - - | 4 |
| Enterodynia - - | 2 | Chlorosis - - | 3 |
| Diarrhœa - - - | 7 | Leucorrhœa - - | 2 |
| Hæmorrhoids - - | 2 | Urticaria - - - | 1 |
| Colica Pictonum - | 3 | Psora - - - | 3 |
| Constipatio - - | 1 | Scabies - - - | 2 |
| Dyspepsia - - | 5 | Phtiriasis - - - | 1 |
| Dropsy - - - | 11 | Herpes - - - | 1 |
| Gravel and Dysury - | 6 | Syphilis - - - | 2 |

Since the last Report, scarcely a day has passed without rain, which has fallen in considerable quantity; at the same time the temperature of the air has been mild, and I have not yet remarked any aggravated symptoms of disease to prevail. On the contrary, the cases which I have seen have been generally less severe than is usual at this season of the year, and I have met with no case of putrid or malignant fever; the principal febrile diseases have been catarrh, measles, scarlatina, and hooping cough.

The proportion of dropsical cases to the other maladies now recorded, is unusually great. One of them is a case of hydrothorax, which has occurred a second time; the patient is a man past the middle period of life; and although he is at present materially relieved by the use of mercury, digitalis, and opium, the symptoms are yet too urgent to admit any rational hopes of recovery. Three of the other patients were affected with anasarca; and the remaining seven labour under ascites. The remote cause, in most of these cases, was evidently the abuse of spirituous liquors, a practice that may justly be regarded as the greatest curse which visits the labouring part of the community; by exhausting their vigour, the powers of the mind are depressed, whilst those of the body are rapidly destroyed; and the poor wretch, at once assailed with misery, disease, and despair, sinks the untimely victim of his own folly. Unfortunately, the women indulge in this fatal custom with as much pertinacity as the men; and not content content with enjoying it themselves, make their little unoffending infants partakers of the poisonous draught. A poor child, whom I attended some time ago for ascites and general emaciation, refused every sort of nourishment, and constantly cried out for gin, of which, from his infancy, I found his mother had been accustomed to allow him a daily portion.

One of the cases of tabes mesenterica was that of a boy aged eight years, who, about seven months ago, began to complain of lassitude and debility, with pain and swelling of the submaxillary glands. A skilful surgeon, who then attended, gave him vinum ferri, which in three weeks not producing any effect, was omitted, and the patient began to take twenty drops of the solution of the muriate of barytes three times a day. He continued this plan three months, at which time I first saw him; the glands in the neck were then less than they had been; the abdomen was tumid, tense, and a slight fluctuation was felt; great sense of pain was expressed on making pressure at the umbilicus; two of the inguinal glands were enlarged; general emaciation was going on; the complexion was wan, and the countenance had an expression of continual pain and irritation; little urine was discharged; and a frequent diarrhœa added to the distress of the patient. With such symptoms, and at so advanced a period of the complaint, little prospect of a cure could be entertained. Three grs. of calomel were now given every other night, and Griffith's myrrh mixture with steel three times a day. These medicines, at first, afforded some relief; the child passed more water, the abdomen was reduced in size, and he seemed more cheerful, and he had less pain. In about a month, however, he began again to grow worse; cicuta was substituted for the other medicines, and was afterwards joined with digitalis; they gave but momentary ease, and in two weeks more death terminated his sufferings. On inspecting the body after death, the appearances were general peritoneal inflammation, with some effusion of serous fluid; coagulable lymph was thrown out, glueing together the small intestines, and uniting them to the parietes of the abdomen; the anterior surface of the liver was attached by adhesive inflammation to the contiguous parts; the mesenteric glands were enlarged, and of various degrees of hardness and size, some of them approaching the state of suppuration, and altogether forming a huge mass. The colon was ruptured, (probably after death)

death) and the gall bladder was entirely empty, and had a whitish appearance. All the other viscera, both of the abdomen and the thorax, were sound.

S. FOTHERGILL.

*Southampton Street, Strand, Dec.* 23, 1806.

